# Gradient Boosting Machine Identified Predictive Variables for Breast Cancer Patients Pre- and Post-Radiotherapy: Preliminary Results of an 8-Year Follow-Up Study

**DOI:** 10.3390/antiox11122394

**Published:** 2022-12-02

**Authors:** Elisabet Rodríguez-Tomàs, Meritxell Arenas, Gerard Baiges-Gaya, Johana Acosta, Pablo Araguas, Bárbara Malave, Helena Castañé, Andrea Jiménez-Franco, Rocío Benavides-Villarreal, Sebastià Sabater, Rosa Solà-Alberich, Jordi Camps, Jorge Joven

**Affiliations:** 1 Unitat de Recerca Biomèdica, Hospital Universitari de Sant Joan, Institut d’Investigació Sanitària Pere Virgili, Universitat Rovira i Virgili, 43201 Reus, Spain; 2 Department of Radiation Oncology, Hospital Universitari de Sant Joan, Institut d’Investigació Sanitària Pere Virgili, Universitat Rovira i Virgili, 43204 Reus, Spain; 3 Functional Nutrition, Oxidation and Cardiovascular Disease Group (NFOC-SALUT), Facultat de Medicina i Ciències de La Salut, Universitat Rovira i Virgili, 43201 Reus, Spain

**Keywords:** biomarkers, breast cancer, cancer recurrence, follow-up, metastatic disease, prognosis, radiotherapy, response to treatment

## Abstract

Radiotherapy (RT) is part of the standard treatment of breast cancer (BC) because of its effects on relapse reduction and survival. However, response to treatment is highly variable, and some patients may develop disease progression (DP), a second primary cancer, or may succumb to the disease. Antioxidant systems and inflammatory processes are associated with the onset and development of BC and play a role in resistance to treatment. Here, we report our investigation into the clinical evolution of BC patients, and the impact of RT on the circulating levels of the antioxidant enzyme paraoxonase-1 (PON1), cytokines, and other standard biochemical and hematological variables. Gradient Boosting Machine (GBM) algorithm was used to identify predictive variables. This was a retrospective study in 237 patients with BC. Blood samples were obtained pre- and post-RT, with samples of healthy women used as control subjects. Results showed that 24 patients had DP eight years post-RT, and eight patients developed a second primary tumor. The algorithm identified interleukin-4 and total lymphocyte counts as the most relevant indices discriminating between BC patients and control subjects, while neutrophils, total leukocytes, eosinophils, very low-density lipoprotein cholesterol, and PON1 activity were potential predictors of fatal outcome.

## 1. Introduction

Breast cancer (BC) is the most frequent type of solid tumor and the second highest cause of cancer death in women [1]. Treatment of BC is hampered by tumors having a wide molecular heterogeneity, with consequences for relapse risk and response to treatment [2]. Several tumor phenotypes have been identified to date (luminal A, luminal B, HER2+, and triple-negative), depending on the putative molecular targets such as estrogen receptors (ER), progesterone receptors (PR), the human epidermal growth factor 2 receptor (HER2) and Ki-67 level. Triple-negative BC (TNBC) is characterized by the lack of expression of these molecular targets. Patients with this BC subtype and ages younger than 40 present an early risk of relapse and a low survival rate compared to other subtypes [3,4]. Adjuvant radiotherapy (RT) is part of the standard BC treatment due to its effects on loco-regional relapse reduction, as well as the improvement in survival for early-stage to locally advanced BC following conservative surgery or post-mastectomy, with or without regional lymph node involvement [5]. However, the efficacy of RT is not definitively curative, and there are some patients with BC who, over time, develop disease progression (DP) [6,7]. In this context, huge efforts have been focused on investigating the causes of treatment resistance and BC progression with the aim to increase the survival and quality of life of these patients [8]. However, results of these efforts are still inconclusive, and this hampers the design of efficient therapeutic strategies or finding biomarkers that identify individuals at high risk of relapse [9]. We, and other research groups, have reported evidence that antioxidant and inflammation systems are associated with the onset and development of BC, and contribute to resistance-to-treatment, and prognosis [10,11]. We have shown that circulating levels of the enzyme paraoxonase-1 (PON1) are decreased in patients with BC, and other types of cancer, compared to the healthy population [12,13,14,15]. This enzyme degrades lipid peroxides in lipoproteins and cells and plays an important antioxidant role in the organism [16]. Moreover, low serum PON1 concentrations post-RT have been associated with metastatic BC [13]. PON1 participates in the control of inflammation, reducing the capacity of macrophages to oxidize low-density lipoproteins, and downregulating the levels of the pro-inflammatory chemokine (C-C motif) ligand 2 (CCL2) [10]. Cytokines play a key role in carcinogenesis because they are involved in processes such as cell growth, differentiation, proliferation, and migration [17,18]. Interleukin-4 (IL-4), interferon-gamma (IFN-γ), and CCL2 promote tumorigenesis during equilibrium and escape stages. High plasma concentrations of these inflammatory markers have been related to tumor metastasis and poor prognosis [19,20]. However, these associations have been identified using traditional statistical methods, which have several limitations in identifying new variables and generating integrative visualizations [21]. In recent years, technological advances such as the employment of machine learning algorithms have been postulated as accurate methods to find predictive variables in cancer [21,22,23]. For example, previous studies have used random forest for BC early detection according to the clinicopathological features of the patients or Gradient Boosting Machine (GBM) to find and classify predictive variables related to prognosis in patients with different types of cancer [24,25,26]. Although this approach is promising, the evidence related to the identification of circulatory parameters capable of stratifying BC patients with and without DP is scarce.

Hence, in the present study, we investigated the clinical evolution of BC patients and the impact of RT administration on the circulating levels of PON1-related variables, cytokines, and standard biochemical and hematological analytes. Moreover, we identified potential biomarkers of BC prognosis using the GBM algorithm.

## 2. Materials and Methods

### 2.1. Study Design and Patient Population

This is a retrospective study in 237 patients with BC attending the Department of Radiation Oncology of the Hospital Universitari Sant Joan de Reus (Reus, Spain) after having undergone surgery for tumor extirpation. Patients were followed-up for the duration of the study of 8 years (from March 2014 to March 2022; median = 6 years; interquartile range = 5–7 years) and segregated for statistical purposes according to whether they had DP. Having DP was defined as presenting local recurrence (LR), loco-regional recurrence (LRR), or distant metastases (DM) (Supplementary Figure S1). Patients with second primary tumors (including contralateral BC) were not included in the DP group. All patients had a Karnofsky Index > 70 and were classified as 0 or 1 on the Eastern Cooperative Oncology Group scale [27]. The exclusion criteria were having previously received RT at the same cancer site, being pregnant, or lactating or unable to follow-up the patient due to a change of city or country residence.

Radiation was administered to the breast, or mastectomy site, with or without nodal irradiation. The radiation schedule was normo-fractionated RT (50 Gy at 2 Gy/day, 5 days/week) or hypo-fractionated RT (40 Gy at 2.67 Gy/day, 5 days/week) to the breast, or mastectomy site. Following whole-breast irradiation, some patients received an additional RT boost at the tumor bed (16 Gy at 2 Gy/day or 13.34 Gy at 2.67 Gy/day, 5 days/week) [28,29]. Thirty percent of the patients received irradiation of regional lymph nodes, depending on existing risk factors [30]. During RT (weekly) and within 90 days post-irradiation, acute toxicity assessments were performed using the criteria of the Radiation Therapy Oncology Group and those of the European Organization for Research and Treatment of Cancer. Subsequently, late toxicity was evaluated using the late effects of normal tissue-subjective objective management analytical (LENT/SOMA) scale [31]. Prior to irradiation, and one month post-RT, blood samples were obtained from all patients for analyses.

As a control group, we used samples of healthy women participating in a population-based study carried out in our geographical area. An accurate description of this population has been published [32]. All participants signed a written informed consent according to the ethical guidelines of the 1975 Declaration of Helsinki. The study was approved by the Institutional Review Board (project code: 14/2017) of the Hospital Universitari Sant Joan de Reus, and written informed consent was obtained from all patients.

### 2.2. Analytical Measurements

Blood samples were obtained before, and one month after, RT administration. Samples for conventional biochemical and hematological analyses were processed immediately. Serum and EDTA-plasma samples for measurement of cytokine and PON1-related variables were stored at −80 °C until batched analyses. Serum PON1 activity was measured as the rate of hydrolysis of phenylacetate at 280 nm, in a 9 mM Tris-HCl buffer, pH 8.0, supplemented with 0.9 mM CaCl_2_, as previously reported [16]. PON1 can hydrolyze multiple substrates, but we chose phenylacetate because it is not toxic, the assay is simple, and is little influenced by PON1 gene polymorphisms [16]. Serum PON1 concentrations were determined using an in-house ELISA with rabbit polyclonal antibodies specific to PON1 [33]. PON1 specific activity was calculated as the ratio between the activity and the concentration, and is a measure of the activity per molecule. Plasma concentrations of CCL2, IL-4, and IFN-γ were measured by ABTS ELISA Development kits (Peprotech, London, UK). Standard biochemical and hematological analyses were performed in a COBAS^®^ 8000 (Roche Diagnostics, Basel, Switzerland) and a Sysmex XN-1000TM (Sysmex GmbH, Norderstedt, Germany) analyzers, respectively.

### 2.3. Statistical Analyses

Standard statistics were performed with the SPSS 24.0 package (SPSS Inc., Chicago, IL, USA). The Student *t*-test (parametric) and the Mann–Whitney U-test (non-parametric) were used to determine differences between any two groups of variables. Kaplan–Meier analyses were performed to estimate the percentage of overall survival (OS), disease-free survival (DFS), and BC-specific survival, using GraphPad Prism 9.0.1 (GraphPad Software, San Diego, CA, USA.

### 2.4. Density Plots, Venn Diagrams, Circular Packaging, and Volcano Plots

The relative frequency of DP development in BC patients along 8 years of follow-up was schematized by density plots. Venn diagrams were used to visualize the relationships between DP subtypes (LR, LRR, and DM), and circular packaging was used to show the hierarchic organization of the organs affected by distant metastatic relapse. Each DP type, or organ affected, was represented as a circle. The size of each circle was proportional to the frequency of the different DP events, or organs affected. Volcano plots were used to highlight associations between the clinico-pathological characteristics of the patients and the measured circulatory analytes.

### 2.5. Two-Dimensional Linear Discriminant Analysis and Heatmap Representations

Two-dimensional Linear Discriminant Analysis (2DLDA) is a supervised dimensionality reduction analysis presented as matrixes and used to identify differences within patient groups [34,35]. Heatmaps were used to visualize significant differences in individual biological markers.

### 2.6. Machine Learning

We used the Scikit-learn package [36] in Python to build machine learning models. We employed GBM (a decision tree method) to identify predictive variables able to maximize the discrimination between patients with DP, patients without DP, and control individuals [24]. The GBM classifier model was trained initially with 80% of the dataset, and later, we tested the remaining 20%.

To evaluate the accuracy of each GBM model we calculated the areas under the curve (AUC) of the Receiver Operating Characteristics (ROC) curves. The Shapley Additive exPlanation (SHAP) method was employed to interpret the optimal GBM model output. This method determines the contribution of each variable to model outputs (termed SHAP value). We depicted the SHAP summary plots as a global bar of the top 5 variables of the chosen prediction model. In these global bar plots, the importance of each feature was taken to be the mean absolute value for that feature over all the given samples.

## 3. Results

### 3.1. Follow-Up of BC Patients

**Figure 1:**
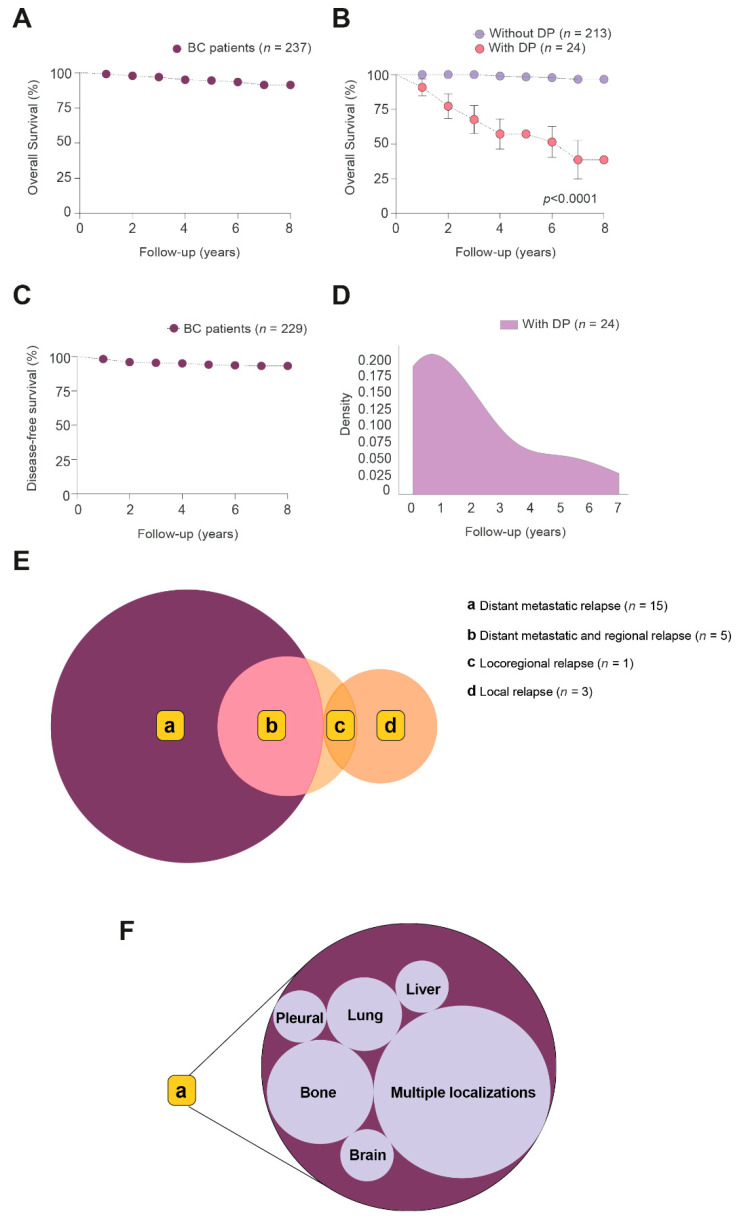
The clinical evolution of patients with breast cancer (BC) after 8 years of follow-up post-RT. (**A**) Kaplan–Meier curve of overall survival (OS) in BC patient groups; (**B**) Kaplan–Meier curve of OS in BC patients segregated with respect to presence or absence of disease progression (DP). (**C**) Kaplan–Meier curve of disease-free survival (DFS) in all BC patients. (**D**) Density plot showing the relative frequency of DP in the 8 years of follow-up post-RT. (**E**) Venn diagram showing the main types of DP. (**F**) Circular packaging showing the organs affected by distant metastatic relapse. Circle sizes are proportional to the frequency of the type of DP, and organs affected.

The 8-year OS of all BC patients was 91.3% (Figure 1A). Of the 237 patients, 24 showed DP over eight years post-RT administration. These patients had a marked OS reduction compared to patients without DP (Figure 1B). The DFS of all BC patients was 93.2% (Figure 1C). Most of the DP events appeared in the first two years (Figure 1D). Metastatic relapse (*n* = 15) was the most frequent event, followed by regional-metastases-and-relapse (*n* = 5), local relapse (*n* = 3), and loco-regional relapse (*n* = 1) (Figure 1E). Among metastatic and metastatic-and-regional-relapse groups, multiple localizations (*n* = 11) were the most frequent metastatic event, followed by involvement of bones (*n* = 4), lung (*n* = 2), liver (*n* = 1), pleural (*n* = 1) and brain (*n* = 1) (Figure 1F). At the conclusion of follow-up, 4 patients of the DP group were alive and disease-free, 7 were alive with disease stabilization, 12 had died of BC, and 1 had died due to heart failure (Supplementary Figure S2A). By contrast, in the patients without DP, 198 patients were alive and disease-free, 8 were alive with a second primary tumor (contralateral BC, endometrial carcinoma, non-melanoma skin cancer, gastric cancer, and large cell lymphoma), 5 had died from other causes (Parkinson’s disease, kidney failure, broncho-aspiration, pulmonary embolism, and second primary tumor), and 2 were lost to follow-up having moved abroad.

### 3.2. Clinico-Pathological Features and Analytical Alterations in BC Patients with and without DP

The baseline characteristics of BC patients segregated according to whether or not they had presented DP are shown in Table 1. Most of the patients who developed DP had been diagnosed with BC at a younger age (median 46 years), compared to those who did not have any event (median 55 years). Their tumors were relatively larger in size, had less positive ER and PR, and a higher percentage of the ki67 index. Neoadjuvant chemotherapy and mastectomy were more commonly employed in DP-risk patient groups. Most of the patients who had died of the BC cancer had had TNBC tumors (Supplementary Figure S2A,B).

Results of all the variables analyzed in patients with and without DP, and pre- and post-RT, are summarized in Supplementary Table S1. When we compared patients with and those without DP, we observed that the former had lower pre-RT levels of hemoglobin, total leukocytes, neutrophils, and lymphocytes, and lower post-RT levels of hemoglobin and triglycerides than the latter. Further, patients without DP had increased hemoglobin and PON1 concentrations, and a decreased total leukocyte, neutrophils, lymphocytes, platelets, IL-4, PON1 activity and PON1-specific activity post-RT. Patients with DP showed similar changes, with only lymphocytes, platelets, IL-4, and PON1 concentration and specific activity reaching statistical significance.

**Figure 2:**
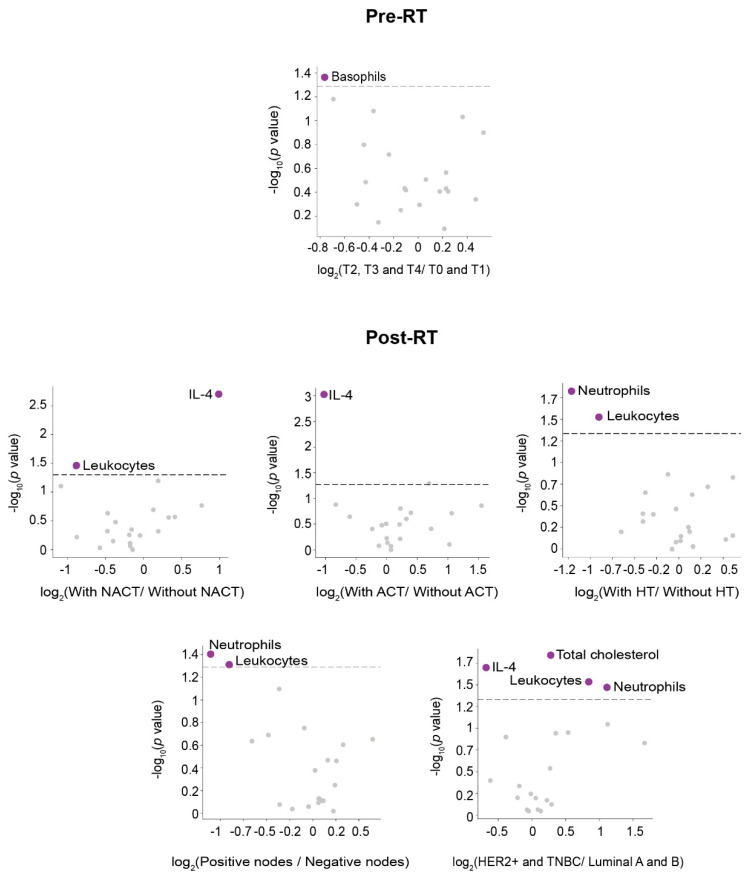
Associations between the clinico-pathological characteristics of patients with disease progression (DP), and the analytical variables pre- and post-radiotherapy (RT). Volcano plots show the significant increase or decrease (shown as purple dots) of specific parameters pre- and post-RT presented in relation to clinico-pathological characteristics, and oncological treatments. Comparisons in the volcano plots were performed using the mean log2-fold change. ACT: Adjuvant chemotherapy; IL-4: Interleukin-4; HER2+: Positive human epidermal growth factor receptor 2; HT: Hormone therapy; NACT: Neoadjuvant chemotherapy; T: Tumor size; TNBC: Triple-negative breast cancer.

**Figure 3:**
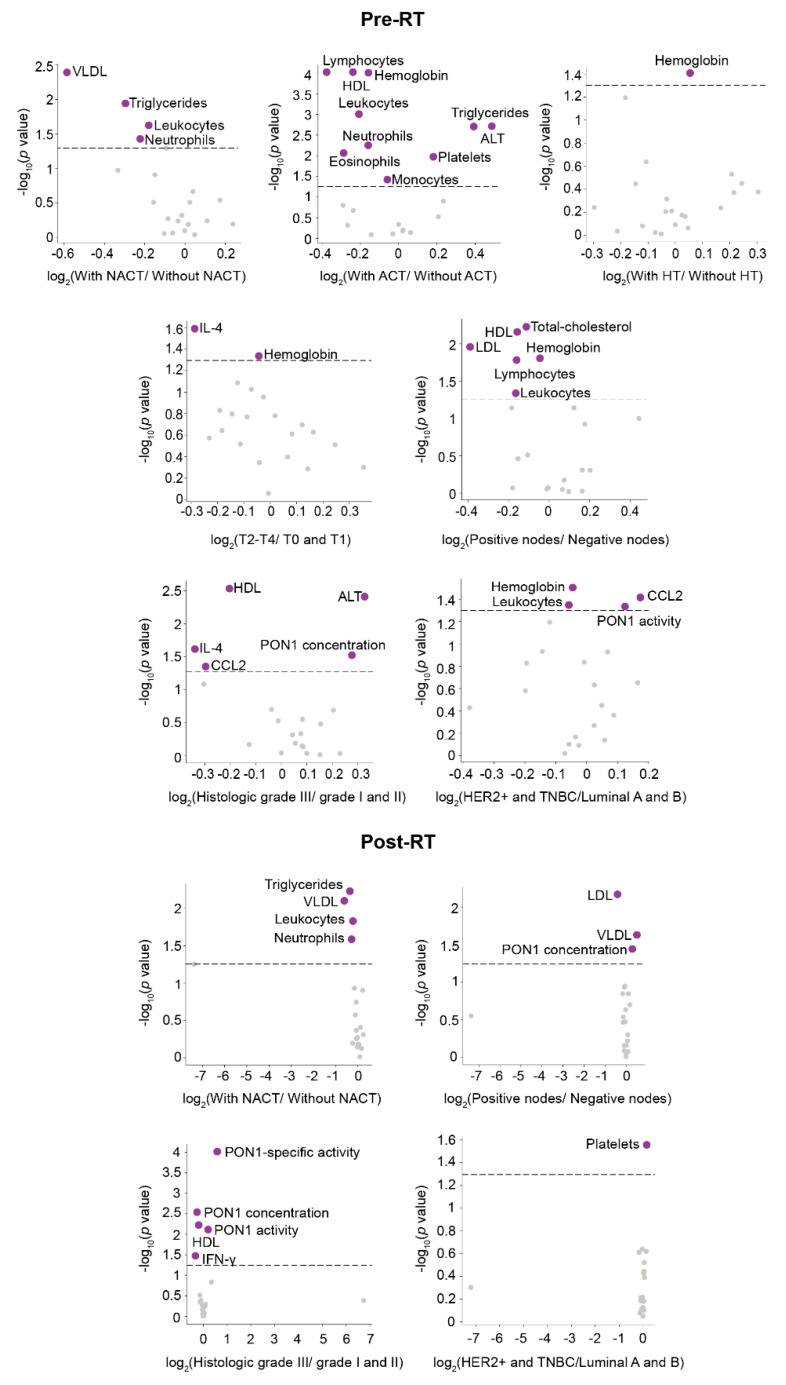
Associations between the clinico-pathological characteristics of patients without disease progression (DP) in relation to the measured analytical variables pre- and post-radiotherapy (RT). Volcano plots show the significant increase or decrease (presented as purple dots) of specific parameters presented as pre- and post-RT in relation to clinico-pathological characteristics of the patients, and their oncological treatments. Comparisons in the volcano plots were performed using the mean log2-fold change. ACT: Adjuvant chemotherapy; ALT: Alanine aminotransferase; CCL2: Chemokine (C-C motif) ligand 2; HDL: High-density lipoproteins; IL-4: Interleukin-4; LDL: Low-density lipoproteins; HER2+: Positive human epidermal growth factor receptor 2; HT: Hormone therapy; IFN-γ: Interferon gamma; NACT: Neoadjuvant chemotherapy; PON1: Paraoxonase-1; T: Tumor size; TNBC: Triple-negative breast cancer; VLDL: Very low-density lipoproteins.

The associations between oncological treatment, tumor characteristics, and the measured circulatory parameters pre- and post-RT are summarized in Figure 2 and Figure 3. The strongest significant differences [–log10(*p*-value > 2.5] were observed pre-RT in patients without DP. Of note is that patients without DP treated with adjuvant chemotherapy (ACT) had lower leukocytes, lymphocytes, monocytes, neutrophils, hemoglobin, high-density lipoprotein (HDL)-cholesterol, and higher alanine aminotransferase (ALT), triglycerides, and platelet levels. Other associations were much weaker.

### 3.3. IL-4 Was the Best Pre-RT Index Predicting the Presence of BC

**Figure 4:**
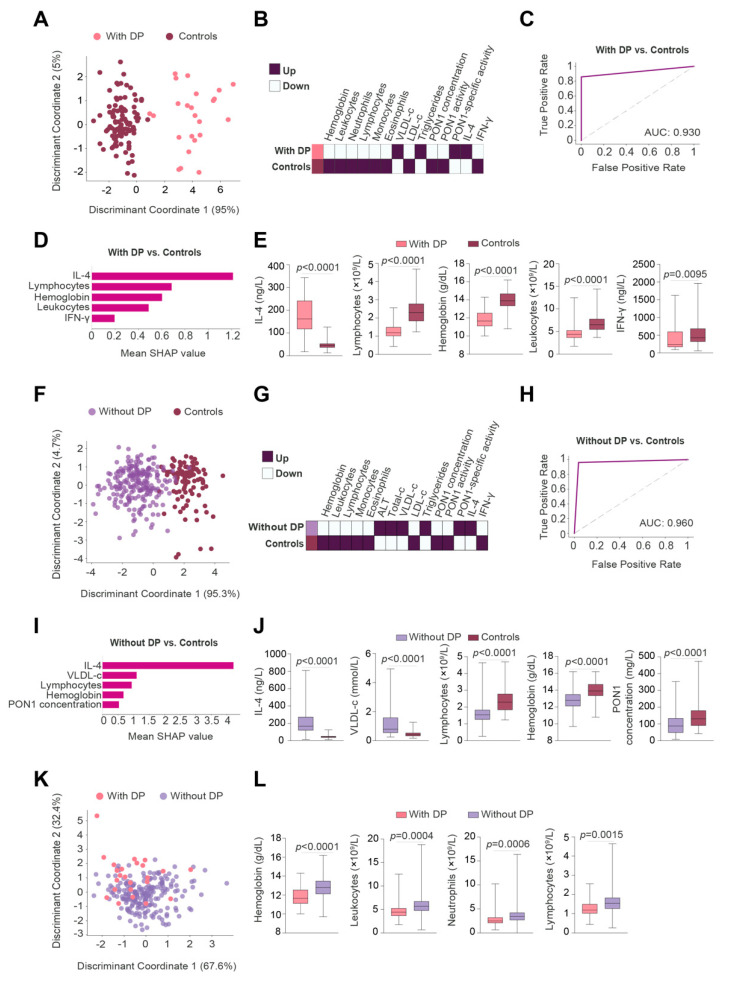
Gradient Boosting Machine (GBM) revealed pre-radiotherapy (RT) IL-4 concentration as the best parameter to discriminate between breast cancer (BC) patients and control subjects. (**A**,**F**,**K**) Two-dimensional linear discriminant analysis of biochemical, inflammatory, and antioxidant markers pre-RT. Each dot represents a BC patient with (pink dots) or without (purple dots) disease progression (DP). Control individuals are presented in brown dots. (**B**,**G**) Heatmap depicts the parameters showing significant differences between BC patients with and without DP vs. control subjects. (**C**,**H**) Receiver operating characteristics (ROC) curve of the models built with the GBM algorithm. (**D**,**I**) The top 5 most important variables are highlighted by mean Shapley Additive exPlanation (SHAP) values, and by box plots. (**E**,**J**,**L**) Box plots highlighted significant differences between patients with and without DP. ALT: Alanine aminotransferase; AUC: Area under the curve; CCL2: Chemokine (C-C motif) ligand 2; IL-4: Interleukin-4; INF-γ: Interferon gamma; LDL-c: Low-density lipoprotein cholesterol; PON1: Paraoxonase-1; VLDL-c: Very low-density lipoprotein cholesterol.

2DLDA showed that the panel of analyzed parameters pre-RT enabled a complete distinction between patients with BC and the control group of subjects, regardless of whether or not the patients presented with DP (Figure 4A,F). Heatmaps showed that patients with or without DP had similar alterations in lipoproteins, white blood cells, IL-4, and PON1-related variables (Figure 4B,G). The main differences were that patients without DP had higher ALT concentrations than control subjects, while patients with DP had lower neutrophil counts than control subjects.

A GBM algorithm was used to identify and classify the best predictive parameters of the panel of analyzed variables enabling us to discriminate between BC patients with PD and the control group of subjects. The AUC of the ROC plot was >0.90 (Figure 4C). The algorithm also identified the five most relevant discriminatory variables: increased IL-4 concentrations followed by decreased lymphocytes, hemoglobin, total leukocytes, and IFN-γ (Figure 4D,E). Similar results were obtained when comparing BC patients without PD versus the control group of subjects, i.e., the most relevant parameters were: increased IL-4 concentrations, followed by increased VLDL-cholesterol, and decreased lymphocytes, hemoglobin, and PON1 concentrations (Figure 4H–J).

2DLDA did not define any clear differences when comparing BC patients with and those without DP (Figure 4K). However, the box plots showed lower concentrations of hemoglobin, leukocytes, neutrophils, and lymphocytes in the DP group (Figure 4L).

Since IL-4 was identified as the most relevant parameter, we wanted to investigate whether differences in lifestyle habits and clinical comorbidities between patients and control individuals influenced the plasma concentrations of this cytokine. Linear regression analyses showed that none of the selected variables was significantly associated with IL-4 concentrations, except for hypothyroidism (Supplementary Table S2).

### 3.4. Lymphocytes Were the Best Post-RT Index Predicting the Presence of BC

**Figure 5:**
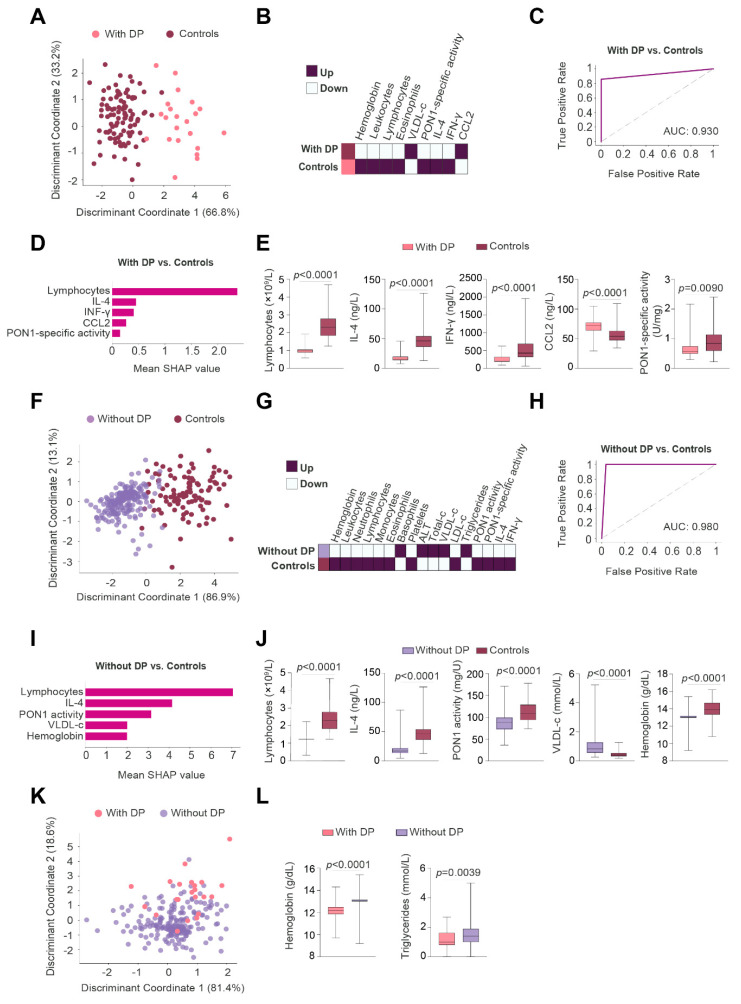
Gradient Boosting Machine (GBM) revealed post-radiotherapy (RT) lymphocyte counts as the best parameter to discriminate between breast cancer (BC) patients and control subjects. (**A**,**F**,**K**) Two-dimensional linear discriminant analysis of biochemical, inflammatory, and antioxidant markers post-RT. Each dot represents a BC patient with (pink dots) or without (purple dots) disease progression (DP). Control subjects are presented as brown dots. (**B**,**G**) Heatmaps depict the parameters showing significant differences between BC patients with and without DP vs. control subjects. (**C**,**H**) Receiver operating characteristics (ROC) curves of the models built with the GBM algorithm. (**D**,**I**) The top 5 most important variables are shown as mean Shapley Additive exPlanation (SHAP) values, and by box plots. (**E**,**J**,**L**) Box plots highlighted significant differences between those patients with and those without DP. ALT: Alanine aminotransferase; AUC: Area under the curve; CCL2: Chemokine (C-C motif) ligand 2; IL-4: Interleukin-4; INF-γ: Interferon gamma; LDL-c: Low-density lipoprotein cholesterol; PON1: Paraoxonase-1; VLDL-c: Very low-density lipoprotein cholesterol.

2DLDA also showed strong post-RT differences in several measured circulatory parameters between patients with BC versus the control group of subjects, regardless of whether the patients presented DP or not (Figure 5A,F). Heatmaps showed similar alterations in both groups of patients (with and without DP) compared to control subjects (Figure 5B,G). The main difference was that patients without DP had higher basophil and lower PON1 activities than the control group of subjects.

The AUC of the ROC plot calculated with the GBM algorithm showed a high diagnostic accuracy in discriminating between BC patients with DP and the control group of subjects (Figure 5C). The five most relevant altered variables in patients with DP were: decreased lymphocytes followed by decreased IL-4, IFN-γ, PON1 specific activity, and increased CCL2 (Figure 5D,E). Similarly, our selected panel of analytes was also efficient in discriminating between patients without events and the control group of subjects (Figure 5H); while the most relevant altered variables were decreased lymphocytes followed by decreased IL-4, PON1 activity, hemoglobin, and increased VLDL-cholesterol (Figure 5I,J).

2DLDA did not identify any major differences when comparing BC patients with, and those without, DP (Figure 5K). However, box plots showed lower hemoglobin and triglyceride concentrations in patients with DP (Figure 5L). Linear regression analyses also showed that the lifestyle habits evaluated, and clinical comorbidities were not significantly associated with lymphocyte concentrations, except for dyslipidemia (Supplementary Table S2).

### 3.5. Relationships between Predictive Variables Pre- and Post-RT, and the Prognosis of Patients Who Developed DP Post-RT

**Figure 6:**
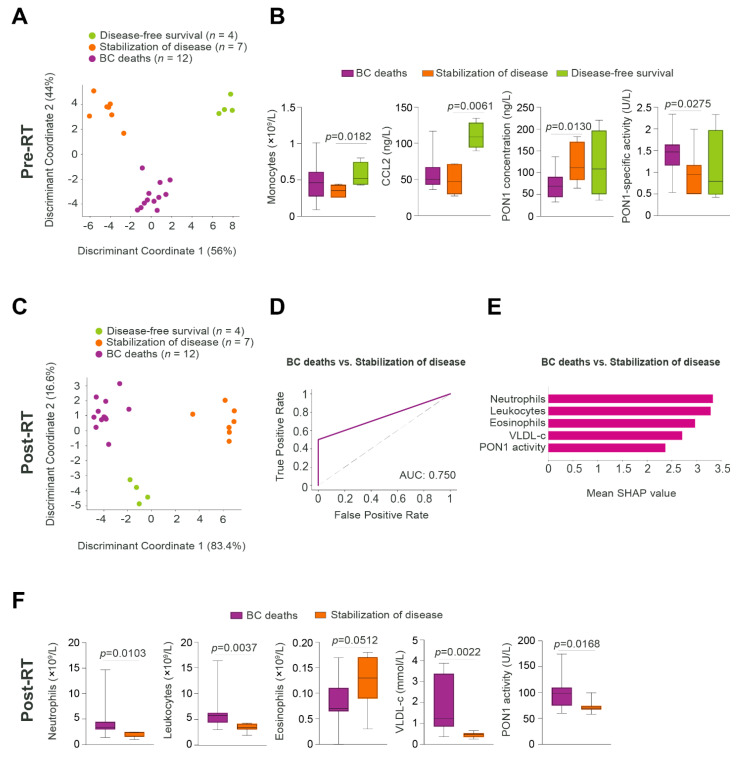
Poor prognosis is related to alterations in inflammatory and oxidative stress parameters. (**A**,**C**) Two-dimensional linear discriminant analysis of biochemical, inflammatory, and oxidative stress markers pre- and post-radiotherapy (RT). Each dot represents a patient with DP according to her status (disease-free survival, DFS, in green, stabilization of disease, SD, in orange and deaths in purple). (**B**) Box plots showing significant differences in the analytical variables according to breast cancer patients’ status. (**D**) Receiver operating characteristics (ROC) curves of the Gradient Boosting Machine algorithm. (**E**,**F**) The top 5 most important variables are highlighted by mean Shapley Additive exPlanation (SHAP) values, and by box plots. AUC: Area under the curve; BC: Breast cancer. PON1: Paraoxonase-1; VLDL-c: Very low-density lipoprotein cholesterol.

We aimed to investigate the relationships between the measured variables and the outcomes in patients who developed any type of DP. We classified these patients according to their current disease status: disease-free survival, stabilization of disease, and BC deaths. 2DLDA pre-RT was able to segregate the three subgroups (Figure 6A). Decreased PON1 concentration and increased PON1 specific activity were the main variables distinguishing between BC deaths and stabilization of disease subgroups. Conversely, increased monocytes and CCL2 were the main variables distinguishing between disease-free survival and the stabilization of disease subgroups (Figure 6B). The 2DLDA post-RT also showed significant differences between the three subgroups (Figure 6C). The GBM model with an AUC of 0.750, indicated that post-RT neutrophils, leukocytes, eosinophils, PON1 activity, and VLDL-cholesterol were the most efficient parameters in the discrimination between stabilization of disease and cancer death (Figure 6D,E). Post-RT, higher values of neutrophils, total leukocytes, PON1 activity and VLDL-cholesterol, and lower eosinophils, were associated with a higher probability of cancer death (Figure 6F). Further linear regression analyses did not highlight any significant associations between lifestyle habits and clinical comorbidities versus neutrophils, except for tobacco use, and diabetes mellitus (Supplementary Table S2).

## 4. Discussion

In the era of personalized medicine, it is crucial to understand the metabolic and molecular bases of pathological processes. Such insights would help design novel therapeutic options and to identify biomarkers of diagnosis, prognosis, and response-to-treatment for better patient management. In BC, it is imperative to identify patients with a high risk of recurrence to avoid general over-treatment that causes pernicious side effects and worsens the patient’s quality of life. Our present study summarizes the RT-induced changes in antioxidants, inflammatory cytokines, and other biological parameters that provide preliminary results on the possible identification of specific biomarkers of outcomes using GBM algorithms.

Our results indicated that patients with DP were younger and had more aggressive tumor characteristics, including larger tumor size, less hormone-receptor positivity, and higher percentage ki67%. Similar results have already been reported, [37,38,39,40] indicating that patients with early recurrence (within 24 months post-surgery) showed poorer prognosis. Moreover, significant risk factors for local recurrence were premenopausal status (younger patients), absence of estrogen receptors, and tumor multi-focality (the growth of multiple tumors in the same area of the breast). Our study supports the well-established concept that younger patients with an aggressive tumor have a high risk of developing DP during the first 2 years post-treatment. Among patients with DP, metastatic relapse in bones was one of the most frequent. Bone is one of the most common sites of metastasis for BC, and once the cancer spreads it is rarely cured [41,42]. BC cells can take control of regulatory pathways for osteoclast differentiation, activation, and survival while promoting bone destruction and tumor growth [43].

We found similar pre- and post-RT alterations in lipoproteins, white blood cells, IL-4, and PON1-related variables in BC patients compared to control subjects, independently of whether they had DP. Moreover, we employed the GBM algorithm to identify the most predictive variables, and we used the SHAP method to interpret the outputs. Previous studies have discussed the use of different machine learning algorithms such as random forest, GBM, and the extreme boosting machine to find predictive variables in patients with BC [44,45]. However, there is no consensus about which algorithm should be used because the accuracy of each model mainly depends on the dataset. Nevertheless, GBM is currently considered the state-of-the-art algorithm for different clinical scenarios [26]. In addition, to understand how the models yielded their predictions, SHAP values have been proposed as the most effective method for a visual explanation of the model and for presenting properties of local accuracy and consistency [46,47]. Indeed, some studies have used SHAP values to select important features for predicting BC molecular subtypes from images [48].

In the present study, GBM identified IL-4 and lymphocytes were the best indices segregating BC patients from control subjects. Data suggest that the pattern and levels of cytokine release are related to cancer onset and development; given that tumors contain a network of pro- and anti-inflammatory cytokines that regulate the clinical evolution of the tumor [49]. Specifically, IL-4 is a γ-chain cytokine secreted by mast cells, T helper 2 (Th2) lymphocytes, eosinophils, and basophils. It is a potent regulator of immunity and cancer development because it promotes the differentiation of naïve CD4+ T cells into the CD4+ Th2 subset and can also influence the function of mature CD8+ T cells [50]. IL-4 and its receptors contribute to the malignant phenotype due to their key role in cell proliferation, migration, and invasion. Blocking IL-4 signaling has been related to apoptotic stimulation of cancer stem-like cells, which suggests inducing IL-4 inhibition as possible therapeutic tools in colon carcinoma [51]. In addition, an enhancement of anti-tumor immunity and delays in tumor progression have been observed in vivo following the administration of neutralizing antibodies against IL-4 [52]. Several clinical studies have found high circulating levels of IL-4 in patients with various types of cancer, as well as a decrease in its levels in patients with complete response following neoadjuvant chemotherapy [53,54,55]. Conversely, lymphocytes are among the body’s most powerful weapons in fighting tumors. The relationship between low levels of peripheral lymphocytes and poor BC prognosis has long been described and is linked to a decrease in the overall immunity of the organism [56]. More recent studies have linked high neutrophils-to-lymphocytes ratio with BC risk [57] and low lymphocyte-to-monocyte ratio as an accurate prognostic marker in BC patients receiving neoadjuvant chemotherapy [58].

Further, GBM analysis in our DP patients indicated that those with the highest post-RT neutrophil counts, and PON1 activities, were most likely to die. The role that neutrophils play in cancer development is currently receiving considerable research attention. Some studies have reported that these leukocytes can adapt to different cancer microenvironments, enhancing the malignancy of cancer cells [59]. In a mouse model of liver metastasis with inflammation, neutrophils were reported to participate in the metastatic process by enhancing cancer cell extravasation, migration, and organ invasion [60]. Conversely, PON1 is one of the main endogenous antioxidant systems protecting cells from the pro-oxidant environment. This enzyme is a lipolactonase that hydrolyzes lipid peroxides in the cytoplasmic and intracellular membrane of the cells, as well as in the circulating lipoproteins [10,16]. PON1 also participates in the innate immune system [61]. Previous studies have observed circulatory alterations in PON1-related variables in patients with different types of cancer [12]. In the current study, lower PON1 levels were found in BC patients, relative to control individuals. Similar results have been reported in earlier studies conducted in patients with BC, and other cancers [13,15,62]. Our current study showed a post-RT increase in PON1 concentration, and a further decrease in PON1 activity. However, patients with DP who died had higher levels of PON1 activity post-RT than patients with stable disease. These results are counter-intuitive and, for which, we lack an adequate explanation. We observed that the levels were much lower than control subjects but were still significantly higher than those DP patients who had not succumbed. One possible explanation is that the higher activity of PON1 in patients who had died reflects a lower efficacy of RT. Thus, the treatment increases oxidative stress, since this the mechanism by which RT combats the tumor. On the other hand, to degrade oxidized lipids, PON1 must covalently bind to them at their active site; the result is that each enzyme molecule that reacts with a peroxide molecule becomes inactivated [16]. Higher oxidative stress implies, then, lower circulating PON1 activity. It is possible that the higher PON1 activities in patients who had died reflects, therefore, a lower production of oxidative stress by RT, and a lower efficacy of the treatment; the consequence being a fatal outcome. In agreement with our results, a recent study reported that patients with prostate cancer recurrence had significantly higher post-RT serum PON1 activity than those who were recurrence-free [63].

We are aware of the limitations of our study, and our conclusions must be considered as preliminary. The most important limitation is that the number of cases with DP is low. Fortunately, with the treatments currently available, the clinical evolution of most BC patients is good, and eventually achieve cured status. Hence, in a study such as ours, we need several more years of data collection (more patients and longer follow-up) before we can draw firmer conclusions. However, it serves a useful purpose to report these preliminary data because we believe they have the potential for advancing our understanding of the biology of BC; the outcome being to encourage other groups to undertake similar research, and the development of personalized medicine.

## 5. Conclusions

The present study provides preliminary evidence that suggests that pre- and post-RT concentrations of IL-4 and total lymphocyte counts, respectively, may be potential biomarkers of BC. Additionally, post-RT concentrations of neutrophils, total leukocytes, eosinophils, and VLDL-c, as well as serum PON1 activity, may be predictors of poor (fatal) outcomes in patients with BC and DP after RT. However, further studies with more protracted follow-up are needed to validate our findings and to identify potential biomarkers that allow us to discriminate between patients with and without DP.

## Figures and Tables

**Table 1:** Clinical characteristics and oncological treatments of breast cancer patients segregated with respect to whether they had disease progression post-radiotherapy.

	With DP (*n* = 24)	Without DP (*n* = 213)	*p* Value
**Clinical characteristics**			
Age at diagnosis (years)	46 (39–55)	55 (47–65)	**0.005**
Alcohol habit (>20 g/day)	-	10 (4.7)	0.278
Smoking habit	5 (20.8)	25 (11.7)	0.203
Hypertension	6 (25)	49 (23)	0.826
Diabetes Mellitus	2 (8.3)	11 (5.2)	0.518
Dyslipidemia	4 (16.7)	51 (23.9)	0.800
Chronic obstructive pulmonary disease	-	7 (3.3)	0.367
Ischemic heart disease	1 (4.2)	6 (2.8)	0.711
Hypothyroidism	-	20 (9.4)	0.116
**Menopause status**			
Premenopausal	9 (37.5)	52 (24.4)	0.164
Peri-menopausal	3 (12.5)	22 (10.3)	0.742
Postmenopausal	12 (50)	139 (65.3)	0.140
Use of oral contraceptives	8 (33.3)	73 (34.3)	0.926
Motherhood	16 (66.7)	162 (76.1)	0.313
**Cancer characteristics**			
**Tumor size (TNM system)**			
T0	2 (8.3)	16 (7.5)	0.885
T1	6 (25)	119 (55.9)	**0.004**
T2	9 (37.5)	60 (28.2)	0.340
T3	3 (12.5)	16 (7.5)	0.393
T4	4 (16.7)	2 (0.9)	**<0.001**
**Nodes (TNM system)**			
N0	10 (41.7)	146 (68.5)	**0.008**
N1	13 (37.5)	49 (23)	**0.001**
N2	3 (12.5)	14 (6.6)	0.286
N3	2 (8.3)	4 (1.9)	0.056
**Metastases (TNM system)**			
M0	24 (100)	(100)	-
M1	-	-	-
**Pathological anatomy of the tumor**			
Ductal carcinoma in situ	-	14 (6.6)	0.195
Invasive ductal carcinoma	22 (91.7)	176 (82.6)	0.257
Lobular carcinoma in situ	1 (4.2)	-	**0.002**
Invasive lobular carcinoma	-	3 (1.4)	0.558
Papillary carcinoma	-	13 (6.1)	0.213
Others	1 (4.2)	7 (3.3)	0.820
**Histological grade**			
I	3 (12.5)	45 (21.1)	0.318
II	11 (45.8)	105 (49.3)	0.747
III	10 (41.7)	63 (29.6)	0.223
**Positive Estrogen receptors**	15 (62.5)	176 (82.6)	**0.018**
**Positive Progesterone receptors**	11 (45.8)	142 (66.7)	**0.043**
**Positive HER2 in tumor biopsy**	7 (29.2)	36 (16.9)	0.139
**Ki67 antigen in tumor biopsy**			
Less than 15%	4 (16.7)	90 (42.3)	**0.015**
15–50%	10 (41.7)	96 (45.1)	0.750
More than 50%	10 (41.7)	27 (12.7)	**<0.001**
**Tumor molecular classification**			
Luminal A	2 (8.3)	74 (34.7)	**0.008**
Luminal B	8 (33.3)	74 (34.7)	0.890
HER2 positive	7 (29.2)	37 (17.4)	0.158
Triple negative	7 (29.2)	28 (13.1)	**0.036**
**Oncological Treatments**			
Surgical procedure			
Lumpectomy	11 (45.8)	179 (84)	**<0.001**
Mastectomy	13 (54.2)	34 (16)	**<0.001**
Neoadjuvant Chemotherapy	15 (62.5)	61 (28.6)	**<0.001**
Adjuvant Chemotherapy	6 (25)	65 (30.5)	0.576
Adjuvant Hormone therapy	14 (58.3)	170 (79.8)	**0.016**
Adjuvant Radiotherapy	24 (100)	213 (100)	-
**Secondary effects of Radiotherapy**			
Epithelitis			
Grade I	13 (54.2)	113 (53.1)	0.917
Grade II	8 (33.3)	93 (43.7)	0.332
Grade III	3 (12.5)	7 (3.3)	**0.033**
Pneumonitis	-	2 (0.9)	0.633

## Data Availability

The data presented in this study are available in the article and Supplementary Materials.

## Supplementary Materials

The following supporting information can be downloaded at: https://www.mdpi.com/article/10.3390/antiox11122394/s1, Figure S1: Flow chart of the retrospective study conducted in patients with breast cancer undergoing radiotherapy; Figure S2: Tumor characteristics are associated with the prognosis of patients with breast cancer (BC); Table S1: Measured biochemical variables in breast cancer (BC) patients segregated with respect to presence and absence of disease progression (DP) post-RT; Table S2: Linear regression analyses of the variables associated with interleukin-4 (IL-4), lymphocytes and neutrophils in breast cancer patients.
