# Supplementary material for: Gradient Boosting Machine Identified Predictive Variables for Breast Cancer Patients Pre- and Post-Radiotherapy: Preliminary Results of an 8-Year Follow-Up Study
Source: Antioxidants (Basel). 2022 Dec 2;11(12):2394. doi: 10.3390/antiox11122394 (PMC9774765; doi:10.3390/antiox11122394)
Supplement: Supplementary file 1 [file antioxidants-11-02394-s001.zip › antioxidants-1907740-supplementary.pdf]

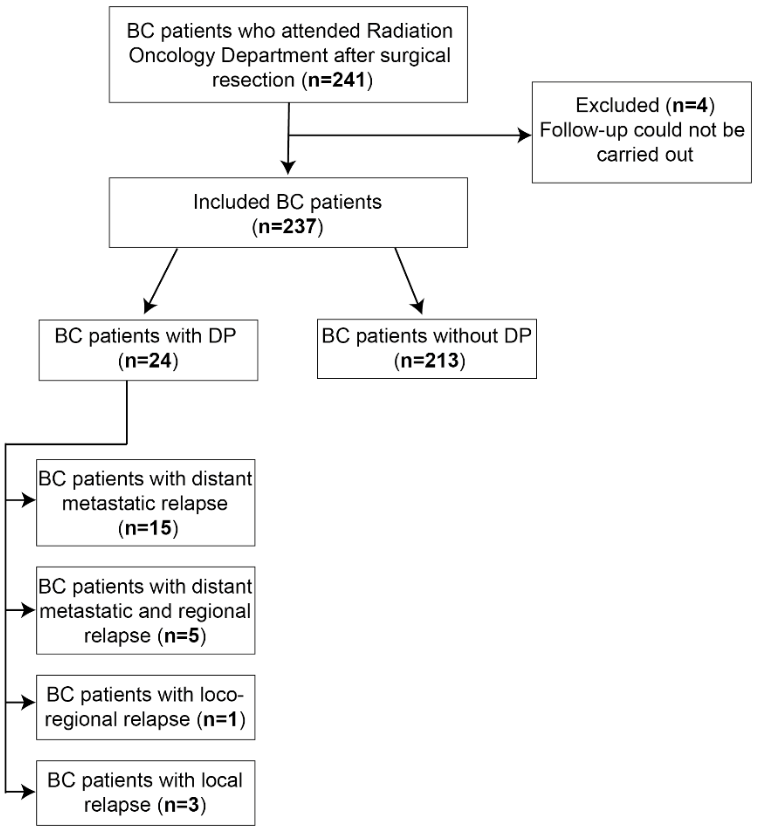

Supplementary Figure S1. Flowchart of the retrospective study conducted in patients with breast cancer who undergo radiotherapy. BC: breast cancer; DP: disease progression.

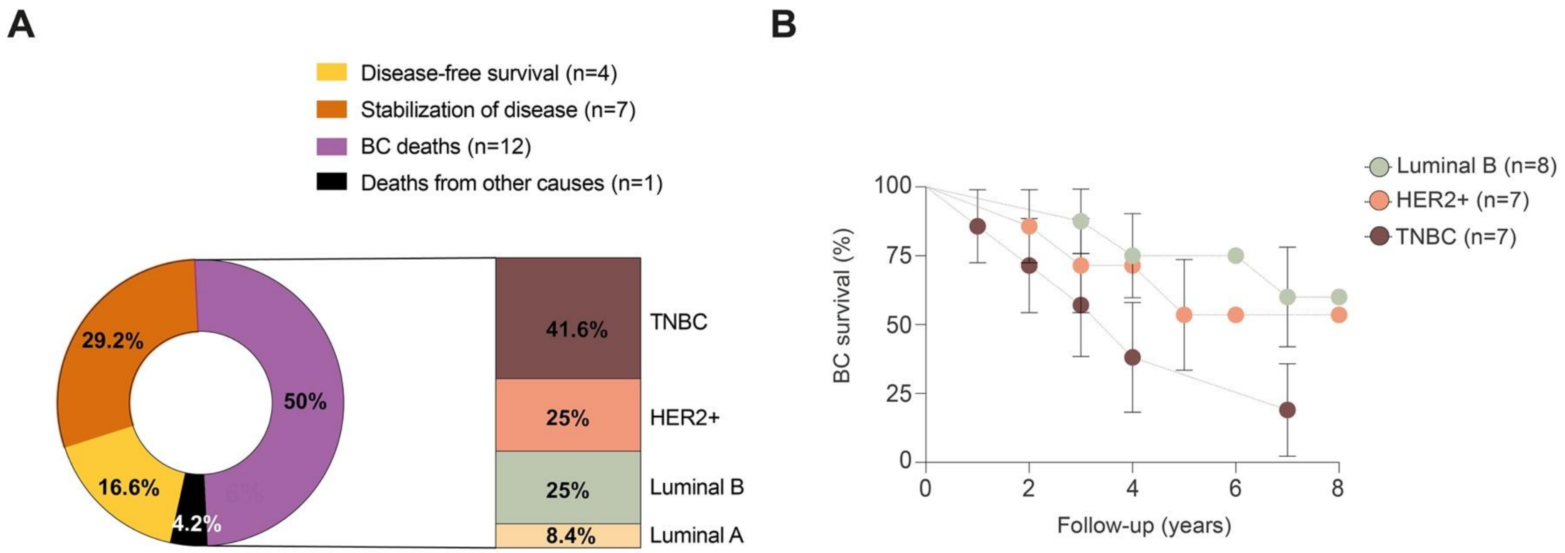

Supplementary Figure S2. Tumor characteristics are associated with the prognosis of patients with breast cancer (BC). The donut chart depicts the status (in percentage) of BC patients 8 years post-RT (A). Figure shows the Kaplan-Meier curve of BC patient survival according to tumor molecular subtypes (B). HER2+: positive human epidermal growth factor receptor 2; TNBC: triple-negative breast cancer.

Supplementary Table S1. Measured biochemical variables in breast cancer (BC) patients segregated with respect to presence and absence of disease progression (DP) post-RT.

	**With DP** (n=24)		**Without DP** (n= 213)		**p value[1]**	**p value[2]**	**p value[3]**	**p value[4]**
	**Pre-RT**	**Post-RT**	**Pre-RT**	**Post-RT**				
**Hemoglobin, g/dL**	11.7 (10.9 - 12.6)	12.2 (11.4 - 13.3)	12.8 (11.9 - 13.7)	13 (12.5 - 13.8)	0.551	**0.002**	**<0.001**	**0.006**
**Leukocytes, x10⁹/L**	4.4 (3.4 - 5.3)	4.4 (3.2 - 6.1)	5.7 (4.3 - 7)	4.8 (3.9 - 6.0)	0.910	**0.007**	**<0.001**	0.583
**Neutrophils, x10⁹/L**	2.4 (2 - 3.3)	2.8 (2.1 - 4.3)	3.4 (2.4 - 4.5)	2.9 (2.4 - 3.8)	0.460	**0.014**	**0.011**	0.741
**Lymphocytes, x10⁹/L**	1.2 (1 - 1.5)	1 (0.7 - 1.2)	1.5 (1.1 - 1.9)	1.2 (0.9 - 1.5)	**0.047**	**0.009**	**<0.001**	0.086
**Monocytes, x10⁹/L**	0.4 (0.3 - 0.5)	0.4 (0.4 - 0.6)	0.4 (0.3 - 0.6)	0.4 (0.3 - 0.5)	0.272	0.348	0.177	0.554
**Eosinophils, x10⁹/L**	0.09 (0.05 - 0.2)	0.1 (0.06 - 0.2)	0.1 (0.07 - 0.2)	0.1 (0.09 - 0.2)	0.210	0.176	0.677	0.139
**Basophils, x10⁹/L**	0.03 (0.01 - 0.03)	0.01 (0.01 - 0.04)	0.02 (0.02 - 0.04)	0.02 (0.01 - 0.03)	0.643	0.853	0.101	0.389
**Platelets, x10⁹/L**	239 (212 - 330.2)	240 (214.2 - 262.5)	243 (199 - 291.2)	221 (181 - 249)	**0.033**	0.602	**<0.001**	0.150
**Total cholesterol, mmol/L**	5.5 (4.9 - 6.1)	5.5 (4.9 - 6.1)	5.6 (4.8 - 6.2)	5.5 (4.8 - 6.1)	0.629	0.803	0.077	0.931
**HDL-cholesterol, mmol/L**	1.4 (1.2 - 1.9)	1.7 (1.4 - 1.9)	1.6 (1.3 - 1.9)	1.6 (1.3 - 1.8)	0.845	0.264	0.148	0.262
**LDL-cholesterol, mmol/L**	0.8 (0.4 - 2.6)	0.6 (0.4 - 2.3)	0.8 (0.5 - 2.4)	0.8 (0.5 - 2.1)	0.744	0.815	0.599	0.876
**VLDL-cholesterol, mmol/L**	2.4 (0.7 - 3.8)	2.7 (0.9 - 3.3)	2.7 (0.9 - 3.5)	2.7 (1 - 3.5)	0.776	0.587	0.155	0.292
**Triglycerides, mmol/L**	1.3 (0.8 - 1.9)	0.9 (0.8 - 1.7)	1.3 (1 - 1.9)	1.4 (0.9 - 1.9)	0.107	0.748	0.732	**0.029**
**AST, µkat/L**	0.3 (0.3 - 0.4)	0.3 (0.3 - 0.4)	0.3 (0.3 - 0.4)	0.3 (0.3 - 0.4)	0.407	0.303	0.409	0.971
**ALT, µkat/L**	0.3 (0.2 - 0.5)	0.3 (0.2 - 0.4)	0.3 (0.2 - 0.4)	0.3 (0.2 - 0.4)	0.468	0.708	0.266	0.912
**PON1 concentration, mg/L**	89 (59.4 - 127)	127.9 (83.5 - 172.6)	88 (48 - 135.5)	125.7 (97.2 - 168.9)	**0.004**	0.774	**<0.001**	0.911
**PON1 activity, U/L**	92.1 (81.7 - 101)	75.4 (66 - 100.9)	97.6 (80.6 - 116.1)	88.1 (65 - 103.2)	**0.035**	0.286	**<0.001**	0.807
**PON1-specific activity, U/mg**	1.2 (0.9 - 1.6)	0.5 (0.4 - 1.3)	1.1 (0.7 - 2)	0.7 (0.4 - 0.9)	**0.027**	0.755	**<0.001**	0.876
**CCL2, ng/L**	56 (43.5 - 84.7)	72.8 (54.9 - 93.7)	51.5 (31.6 - 75.2)	62 (28.7 - 83.6)	0.332	0.115	0.368	0.137
**IL-4, ng/L**	162.6 (116.9 - 242.6)	15.1 (12.9 - 21.5)	168.4 (118.8 - 283.7)	17.1 (12.6 - 24.7)	**<0.001**	0.342	**<0.001**	0.626
**IFN-γ, ng/L**	238.9 (164.6 - 601.3)	192.5 (172.5 - 396.8)	307.9 (213.4 - 678)	279.5 (151.7 - 509.4)	0.093	0.252	0.444	0.387

Values are presented as medians and interquartile range.

[1] Comparison between pre- and post-RT in patients with DP

[2] Comparison between pre-RT without DP and pre-RT in patients with DP

[3] Comparison between pre- and post-RT without DP

[4] Comparison between post-RT without DP and post-RT in patients with DP

AST: aspartate aminotransferase; ALT: alanine aminotransferase; HDL: high-density lipoproteins; LDL: low-density lipoproteins, VLDL: very low-density lipoproteins; PON1: paraoxonase-1; CCL2: C-C Motif Chemokine Ligand 2; IL-4: interluekine-4; IFN-γ: interferon gamma

Supplementary Table S2. Linear regression analyses of the variables associated with interleukin-4 (IL-4), lymphocytes and neutrophils in breast cancer patients.

[A] **IL-4 concentration pre-RT; ng/L**	**B**	**95% CI of B**	**p value**
Constant	88.086	-120.261 - 296.433	0.406
Alcohol habit	9.513	-22.647 - 41.674	0.560
Smoking habit	-9.291	-37.637 - 19.055	0.519
Diabetes Mellitus	58.274	-19.612 - 136.16	0.142
Hypertension	-23.764	-65.941 - 18.414	0.268
Dyslipidemia	4.741	-34.297 - 43.78	0.811
Ischemic heart disease	51.12	-31.694 - 133.934	0.225
Chronic obstructive pulmonary disease	35.802	-39.635 - 111.239	0.351
Menopausal status	0.026	-27.142 - 27.194	0.998
Hypothyroidism	-72.493	-127.623 - -17.364	0.010
[B] **Lymphocytes concentration post-RT; x10^9/L**			
Constant	2.401	1.382 - 3.421	<0.001
Alcohol habit	-0.037	-0.18 - 0.105	0.605
Smoking habit	-0.07	-0.189 - 0.048	0.242
Diabetes Mellitus	0.008	-0.373 - 0.388	0.968
Hypertension	0.181	-0.021 - 0.383	0.078
Dyslipidemia	-0.244	-0.442 - -0.047	0.016
Ischemic heart disease	-0.232	-0.617 - 0.153	0.235
Chronic obstructive pulmonary disease	-0.169	-0.52 - 0.182	0.342
Menopausal status	-0.111	-0.233 - 0.011	0.073
Hypothyroidism	0.044	-0.219 - 0.308	0.739
[C] **Neutrophils concentration post-RT; x10^9/L**			
Constant	7.944	3.745 - 12.144	<0.001
Alcohol habit	0.446	-0.142 - 1.034	0.135
Smoking habit	-0.501	-0.989 - -0.013	0.044
Diabetes Mellitus	-2.906	-4.473 - -1.34	<0.001
Hypertension	0.334	-0.498 - 1.166	0.428
Dyslipidemia	-0.576	-1.39 - 0.237	0.163
Ischemic heart disease	0.829	-0.757 - 2.415	0.303
Chronic obstructive pulmonary disease	-0.337	-1.783 - 1.109	0.645
Menopausal status	-0.096	-0.598 - 0.406	0.706
Hypothyroidism	0.424	-0.663 - 1.511	0.441

[A] Model summary: r^2 = 0.203; p value= 0.413
[B] Model summary: r^2 = 0.368; p value = 0.041
[C] Model summary: r^2 = 0.402; p value = 0.012
RT: Radiotherapy.
